# β1-Integrin and Integrin Linked Kinase Regulate Astrocytic Differentiation of Neural Stem Cells

**DOI:** 10.1371/journal.pone.0104335

**Published:** 2014-08-06

**Authors:** Liuliu Pan, Hilary A. North, Vibhu Sahni, Su Ji Jeong, Tammy L. Mcguire, Eric J. Berns, Samuel I. Stupp, John A. Kessler

**Affiliations:** 1 Department of Neurology, Northwestern University, Chicago, Illinois, United States of America; 2 Department of Biomedical Engineering, Northwestern University, Evanston, Illinois, United States of America; 3 Department of Materials Science and Engineering, Northwestern University, Evanston, Illinois, United States of America; 4 Department of Chemistry, Northwestern University, Evanston, Illinois, United States of America; 5 Department of Medicine and Institute for BioNanotechnology in Medicine, Northwestern University, Chicago, Illinois, United States of America; University of Toronto, Canada

## Abstract

Astrogliosis with glial scar formation after damage to the nervous system is a major impediment to axonal regeneration and functional recovery. The present study examined the role of β1-integrin signaling in regulating astrocytic differentiation of neural stem cells. In the adult spinal cord β1-integrin is expressed predominantly in the ependymal region where ependymal stem cells (ESCs) reside. β1-integrin signaling suppressed astrocytic differentiation of both cultured ESCs and subventricular zone (SVZ) progenitor cells. Conditional knockout of β1-integrin enhanced astrogliogenesis both by cultured ESCs and by SVZ progenitor cells. Previous studies have shown that injection into the injured spinal cord of a self-assembling peptide amphiphile that displays an IKVAV epitope (IKVAV-PA) limits glial scar formation and enhances functional recovery. Here we find that injection of IKVAV-PA induced high levels of β1-integrin in ESCs in vivo, and that conditional knockout of β1-integrin abolished the astroglial suppressive effects of IKVAV-PA in vitro. Injection into an injured spinal cord of PAs expressing two other epitopes known to interact with β1-integrin, a Tenascin C epitope and the fibronectin epitope RGD, improved functional recovery comparable to the effects of IKVAV-PA. Finally we found that the effects of β1-integrin signaling on astrogliosis are mediated by integrin linked kinase (ILK). These observations demonstrate an important role for β1-integrin/ILK signaling in regulating astrogliosis from ESCs and suggest ILK as a potential target for limiting glial scar formation after nervous system injury.

## Introduction

After injury to the nervous system, astrocytes undergo a series of morphologic and molecular changes that facilitate sealing of the blood brain barrier and functional recovery [10], [11], [42]. However, there is also an increase in the number of astrocytes and formation of a glial scar that significantly impedes axonal regeneration [10], [48], [50], [54]. New astrocytes are generated after spinal cord injury (SCI) both by ependymal stem cells (ESCs) as well as by preexisting astrocytes [2]. Several signaling pathways are known to be involved in the generation of astrocytes after SCI [18], [37], but the precise molecular mechanisms regulating astrogliosis are not known.

Injection into an injured spinal cord of a self-assembling peptide amphiphile (PA) that contains an IKVAV epitope (IKVAV-PA) markedly reduced glial scar formation after SCI [54]. IKVAV-PA also profoundly suppressed astrocytic differentiation of cultured neural stem/progenitor cells (NSCs) [47]. The IKVAV sequence is an important neuroactive site on the laminin A chain that can mimic the effects of laminin-1 on neurite outgrowth [36], [46], [53]. Some of the effects of the IKVAV epitope are mediated via the β1-integrin receptor subunit [14] which is the most ubiquitously expressed integrin subunit. This suggested the possibility that β1-integrin signaling might be involved in astrogliosis after SCI.

The integrin family of cell surface receptors is an important link between cells and the extracellular matrix (ECM) and acts both to anchor the cell surface to the ECM and to initiate a variety of intracellular signaling events. Integrin signaling is complex. Integrin heterodimer formation can be induced both by extracellular ligand binding and by cytoplasmic signaling [45], resulting in a conformational change into an activated form. Integrin activation eventually induces intracellular signaling through assembly of a signaling complex at its cytoplasmic tail [7]. Integrins play multiple roles during the development of the nervous system [34]. β1-integrin is highly expressed by neural stem/progenitor cells (NSCs) and in fact has been used as a cell surface marker for NSC enrichment [15], [40]. β1-integrin signaling is an important pathway by which NSCs respond to cues from the ECM to regulate both survival and proliferation [25]. Higher levels of β1-integrin expression by cultured NSCs correlate with a higher capability for self-renewal mediated via the MAPK cascade [8].

Although β1-integrin signaling influences astrocytic morphology and gene expression, it does not alter astrocyte proliferation [43]. We therefore asked whether the effects of IKVAV-PA in limiting the glial scar might be mediated by interactions with β1-integrin expressed by ESCs, and what downstream signaling pathways might be involved. We report a series of observations that demonstrate an important role for β1-integrin/ILK signaling in regulating astrocytic differentiation of ESCs and suggest ILK as a potential target for limiting glial scar formation after nervous system injury.

## Materials and Methods

### Animals

Timed pregnant CD1 mice, 129 SvJ mice and Long Evans rats were supplied by Charles River Laboratories (Wilmington, MA). β1 integrin floxed mice were supplied by Jackson Laboratory (strain name: B6;129-Itgb1tm1Efu/J).

### Cell Culture reagents

Neurosphere growth media were made from supplementing DMEM/F12 (Gibco/Invitrogen) with N2 Supplement (Gibco/Invitrogen) and B27 Supplement (Gibco/Invitrogen), along with Pen/Strap/Glut 100x Mix (Gibco/Invitrogen). ILK inhibitor Cpd 22 was purchased from Millipore and dissolved in DMSO. IKVAV-PA and VVIAK-PA were made by the laboratory of Dr. Samuel Stupp (Northwestern University, IL, US).

### Generation of progenitor cell neurospheres and differentiation cultures

To isolate NSCs from spinal cord, the spinal cord of postnatal day 1 mice were mechanically dissociated using razor blades along with pipetting and grown in serum-free neurosphere growth medium with EGF (20 ng/ml, human recombinant, BD) plus bFGF (20 ng/ml, human recombinant, Millipore) supplemented with Heparin (Sigma) for 7 days, to generate neurospheres. Primary spheres were grown for 7 days *in vitro* (DIV), and then passaged by dissociating with 0.05% trypsin (Invitrogen) for 2 minutes followed by incubation with a soybean trypsin inhibitor (Sigma), a 5- minute spin, and repeated trituration. Secondary spheres were grown for an additional 5–7 DIV and used for subsequent studies.

To isolate NSCs from SVZ, the lateral ganglionic eminences of postnatal day 1 mice were dissociated and grown in serum-free neurosphere growth medium with EGF (20 ng/ml, human recombinant, BD) for 5 days, as previously described, to generate neurospheres [30], [56]. Primary spheres were grown for 3–4 days *in vitro* (DIV), and then passaged by dissociating with 0.05% trypsin (Invitrogen) for 2 minutes followed by incubation with a soybean trypsin inhibitor (Sigma), a 5 minute spin, and repeated trituration. Secondary spheres were grown for an additional 3–4 DIV and used for subsequent studies.

For differentiation studies, neurospheres were dissociated and plated at a density of 1×10^4^ cells/cm^2^ onto poly-D-lysine-coated (PDL, Sigma) coverslips additionally coated with Laminin (Roche) within 24-well culture plates, and then grown for 7 DIV in neurosphere growth medium with low concentration EGF(1 ng/ml or 0.2 ng/ml).

### Immunochemistry of cultures

Prior to PFA fixation, O4 antibody (mouse IgM, Chemicon) was added to cells for 30 minutes at 4°C. 10 µM thymidine analog 5-Ethyl-2′-deoxyuridine (EdU, invitrogen) was added to the cells for 12 hours. Fixed coverslips were incubated with primary antibodies at 4°C overnight in blocking media (0.2% Triton with 1% BSA in PBS). Antibodies were as follows: GFAP (Rabbit, Dako); βIII-tubulin (mouse IgG2b, Sigma); β1 integrin (Rat, Millipore); β1 integrin, activated, HUTS-4 (Mouse IgG2b, Millipore); phosphore-ILK, Ser246 (Rabbit, Millipore); ILK (Rabbit, Millipore); Sox2 (Goat, Santa Cruz). Primary antibodies were visualized with Alexa 647- (infrared) Alexa 555/594- (red), Alexa 488- (green) and Alexa 350- (blue) conjugated secondary antibodies (Invitrogen). Cells were counted in 26 alternate fields of each coverslip and verified in a minimum of three independent experiments.

### Western Blot analysis

Cells were lysed in T-PER Tissue Protein Extraction Reagent (Pierce, Thermo Scientific), supplemented with complete protease and phosphatase inhibitor cocktail (Roche). After removal of cellular debris by centrifugation at 13200 g for 5 min at 4°C, the protein concentration of the lysate was determined using the Bio-Rad Protein Assay standardized to bovine serum albumin. The indicated amounts of cell lysate were resolved by sodium dodecyl sulfate–polyacrylamide gel electrophoresis (SDS–PAGE) and electro-transferred onto nitrocellulose membranes (Bio-Rad). The following primary antibodies were used at 1∶1000 dilution unless otherwise noted: pFAK (Rabbit, Cell Signaling); FAK (Rabbit, Cell Signaling); GFAP (Rabbit, Dako); ILK (Rabbit, Millipore); GAPDH (Mouse IgG, Millipore); pAkt (Rabbit, Cell Signling); Akt (Rabbit, Cell Signaling). Horseradish peroxidases (HRP)-conjugated secondary antibodies (Santa Cruz) were used at 1∶2000 dilutions. Detection was carried out using SuperSignal West Femto Maximum Sensitivity Substrate detection system (Pierce). Immunoblots were stripped and re-probed using Restore Western Blot Stripping Buffer (Pierce).

### RT-qPCR (reverse transcriptase-quantitative polymerase chain reaction)

RNA was harvested from plated cells using Trizol (Invitrogen) and RNAqueous-Micro kit (Ambion). 500 ng of RNA was used to generate cDNA using oligo-dT primers (Thermoscript RT-PCR kit, Invitrogen). Real-time PCR was performed using SybrGreen Master Mix (Applied Biosystems). Three replicates were run for each cDNA sample with the test and control primers. An amplification plot showing cycle number versus the change in fluorescent intensity was generated by the Sequence Detector program (Applied Biosystems).

### Retrovirus production and infection

The β1 integrin constructs were cloned into the pCLE-IRES-EGFP retroviral vector and the retrovirus production is as previously described [4]. 293 FT cells were transfected with pCLE retroviral and pCMV-VSVG helper plasmids using Lipofectamine 2000 transfection reagent (Invitrogen). The supernatant from the cells was collected on day 2, 4 and 7 post transfection, concentrated, tittered and then ∼10^7^ viral particles were used to infect 2.5 million cells for 48 hours in sphere forming media (containing 20 ng/ml EGF). Cells were then FACS sorted for GFP and then either used immediately for RNA isolation or plated on PDL-laminin as described above.

### Thymidine analog labeling

For 5-ethynyl-2′-deoxyuridine (EdU) (Invitrogen) labeling of neurospheres in culture, cells were incubated in neurospheres media containing 10 µM EdU for 12 h and then processed for EdU detection according to the protocol of the manufacturer (Click-IT Flow Cytometry Assay kit; Invitrogen).

### Generation of mutated ILK, b1integrin constructs

Constitutive active ILK expressing construct ILK_S343D were generated by point mutation using QuikChange II XL Site-Directed Mutagenesis Kit (Agilent Technologies). Mutation backbone template was mouse ilk expressing vector ordered from OriGene (ilk (NM_010562) Mouse cDNA ORF Clone). Mutation primers were designed as follows: 5′-GGCTGATGTTAAGTTTGATTTCCAGTGCCCTGGG-3′ and 5′-CCCAGGGCACTGGAAATCAAACTTAACATCAGCC-3′. Kinase dead ILK expressing construct ILK_S343A were generated using mutation primers designed as follows:

5′-GCTGATGTTAAGTTTGCTTTCCAGTGCCCTG-3′.

5′-CAGGGCACTGGAAAGCAAACTTAACATCAGC-3′.

### Mouse spinal cord injury and PA injection

All animal procedures were performed in accordance with the Public Health Service Policy on Humane Care and Use of Laboratory Animals and all procedures were approved by the Northwestern University Institutional Animal Care and Use Committee. Female 129 SvJ mice (10 weeks of age) or female Longs Evans Rats (8 weeks of age) were anesthetized by inhalation of 2.5% isoflurane anesthetic in 100% oxygen administered by a VetEquip Rodent anesthesia machine. A T11 vertebral laminectomy was performed to expose the spinal cord. The spinal cord was injured using an IH-0400 Spinal Cord impactor (Precision Systems) with a 1.25 mm tip with 70 Kdynes of force and 60 s of dwell time for mice and with a 2.50 mm tip with 185 Kdynes of force and 60 s of dwell time for rats. Skin was sutured using AUTOCLIPs (9 mm; BD Biosciences). For postoperative care, animals were kept on a heating pad for 24 h to maintain body temperature. Both mice and rats were given Buprenex (2.5 mg/kg, s.c.) and Baytril (5 mg/kg, s.c.) to minimize discomfort and infection. Bladders were manually expressed twice daily. A 5d Baytril treatment course (5 mg/kg daily, s.c.) was started in the event of hematuria.

PA (1% aqueous solution) or vehicle was injected 48 h after SCI using borosilicate glass capillary micropipettes (Sutter Instruments, Novato, CA) (outer diameter, 100 µm). The capillaries were loaded onto a Hamilton syringe using a female luer adaptor (World Precision Instruments, Sarasota, FL) controlled by a Micro4 microsyringe pump controller (World Precision Instruments). The amphiphile was diluted 1∶1 with a 580 µM solution of glucose just before injection and loaded into the capillary. Under Avertin anesthesia, the autoclips were removed and the injury site was exposed. The micropipette was inserted to a depth of 750 µm measured from the dorsal surface of the cord for mice and a depth of 1250 µm measured for rats, and 2.5 µl for mice or 5.0 µl for rats of the diluted amphiphile solution or vehicle was injected at 1 µl/min. The micropipette was withdrawn at intervals of 250 µm to leave a trail (ventral to dorsal) of the PA in the cord. At the end of the injection, the capillary tip was left in the cord for an additional 1 min, after which the pipette was withdrawn and the wound closed. For all experiments, the experimenters were kept blinded to the identity of the animals.

### Animal perfusions, tissue processing and immunohistochemistry

Animals were killed using an overdose of halothane anesthesia and transcardially perfused with 4% paraformaldehyde in PBS. The spinal cords were dissected and fixed for 2 h with 4% paraformaldehyde and subsequently overnight in 30% sucrose in PBS. The spinal cords were then frozen in Tissue-Tek embedding compound and sectioned on a Leica (Deerfield, IL) CM3050S cryostat.

20 µm thick frozen sections were cut on a leica (CM3050S) Cryostat and collected on Superfrost Bond Rite slides (Richard Allen Scientific). Every fifth section was placed on the same slide such that each adjacent section was 80 µm away from its neighbor. Four sections were placed on each slide and hence the width of the frozen cord spanned on each slide was 240 µm. We roughly averaged about 20 slides per cord. Sections were processed for immunohistochemistry in the same manner as described for the cells. Primary antibodies used were: GFAP (Sigma, mouse IgG1 1∶500), β1integrin (Millipore, rat IgG, 1∶500), HUTS-4 (Millipore, mouse IgG1 1∶500).

### Statistical analyses

Student’s unpaired t test was used for all two group comparisons. Analysis of variance (ANOVA) with the Bonferroni post hoc test was used for all multiple group experiments. P values<0.05 were deemed significant. Values in graphs are shown are Mean ±S.E.M.

## Results

### Knockout of β1-integrin from cultured ESCs increases astrocytic differentiation

**Figure 1:**
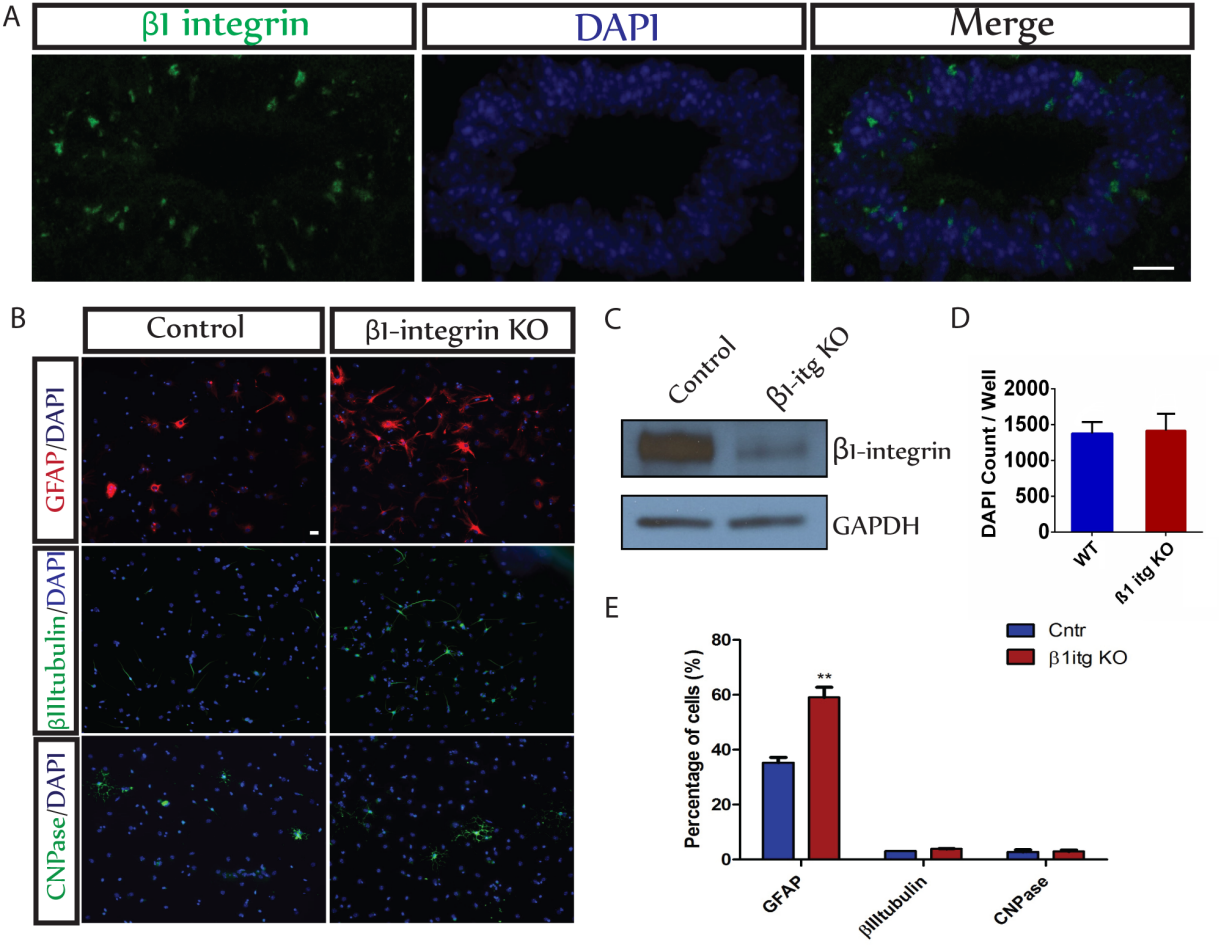
β1-integrin suppresses generation of astrocytes by NSCs derived from the spinal cord. A. Ependymal NSCs in adult mouse spinal cord express β1-integrin. B. NSCs derived from spinal cord are able to differentiate into astrocytes (GFAP -red), neurons (βIIItubulin-green) and oligodendrocytes (CNPase-green). β1-integrin Knock-Out (KO) increases astrocyte differentiation without altering neuronal or oligodendroglial differentiation. *Scale bar = 20 µm*. C. Western blot analysis shows depletion of β1 integrin protein levels in β1-itg KO NSCs derived from the spinal cord. D. Quantification of total cell numbers (DAPI) in the cultures of control and β1-integrin null cells analyzed for lineage commitment (E). There was no difference in overall cell numbers (n = 12, p = 0.89). Values are means ± SEM. E. Quantification of cell numbers of different lineages shows a significant increase (**p≤0.004) in astrocytes (67% of increase) in cultures of β1 integrin null cells at 7DIV without any change in neuronal or oligodendroglial differentiation.

Immunocytochemical examination of adult spinal cord revealed that β1-integrin is expressed predominantly in the ependymal zone (Figure 1A) where ESCs reside [32]. ESCs are able to form neurospheres and can generate all three neural lineages, neurons, astrocytes and oligodendrocytes (Figure 1B and Meletis et al, 2008 [32]). To assess the role of β1-integrin signaling in this process, we isolated and cultured ependymal cells from β1-integrin^flx/flx^ mice and ablated out β1-integrin by infecting cells with Adeno-Cre-GFP virus or a control Adeno-GFP virus. Two days after virus infection, neurospheres were dissociated, sorted by fluorescence activated cell sorting (FACS) to select virus infected cells, and grown again as neurospheres. Western blot analysis confirmed the depletion of β1-integrin in the Adeno-Cre-GFP treated cells (Figure 1C). One week later the cells were dissociated, plated in differentiating conditions for 7 days, and analyzed immunocytochemically (Figure 1B, 1D, 1E). The total number of cells did not differ between control and β1 integrin null cultures (Figure 1D). Control ESCs generated a large number of GFAP-expressing astrocytes (35.3±3.3%of the cells) and smaller numbers of βIIItubulin-immunoreactive neurons (3.3±0.1%) and CNPase-expressing oligodendrocytes (∼3.0±1.3%) (Figure 1B, 1E). The remainder of the cells remained undifferentiated. Knockout of β1 integrin significantly increased the number of GFAP^+^ cells by 67% (to a total amount of 59.1±3.7% of the cells; p≤0.004) without altering the numbers of neurons or oligodendroglia (Figure 1B, 1E). This suggests that β1-integrin signaling suppresses astrocytic differentiation by ESCs.

**Figure 2:**
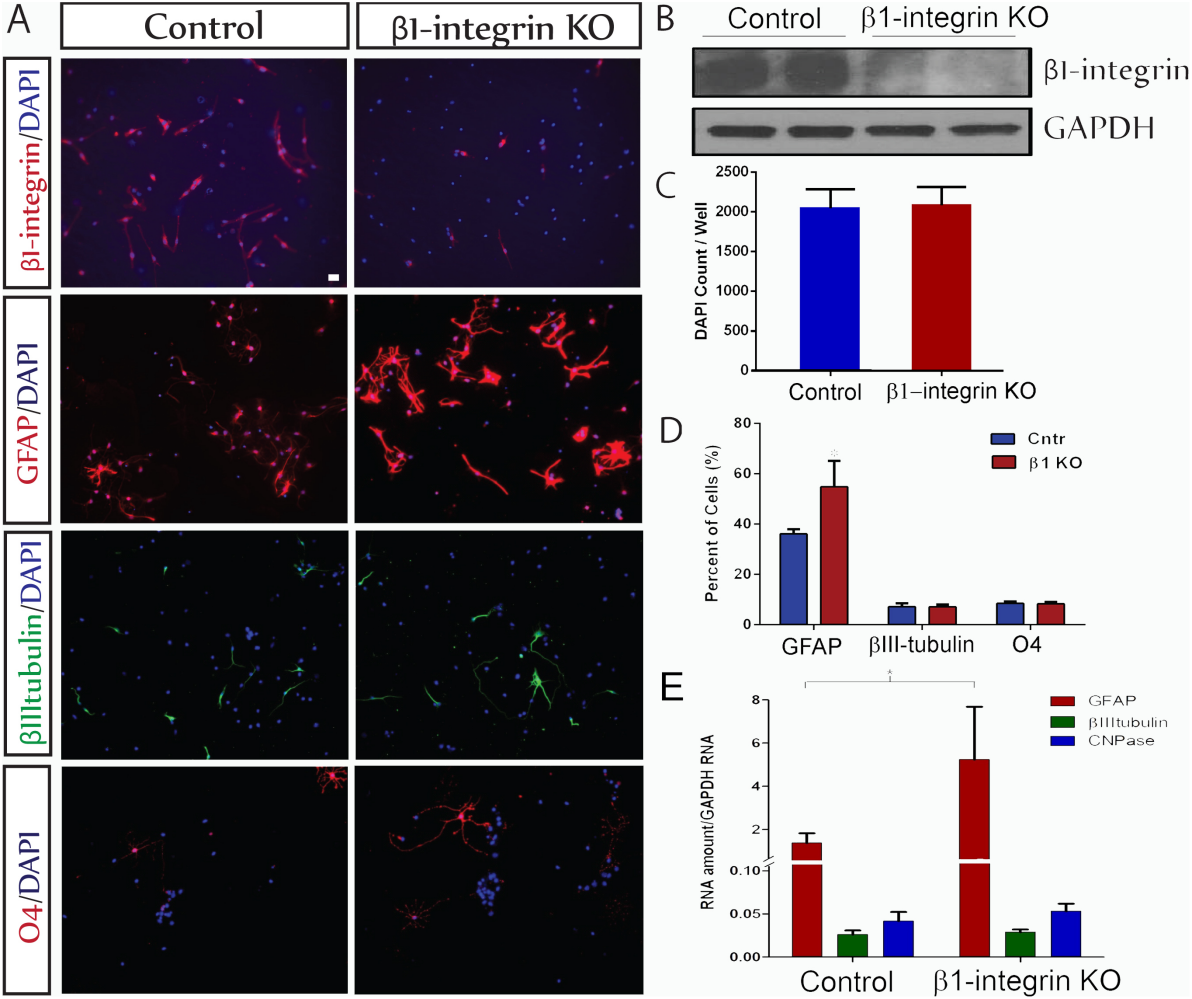
β1-integrin suppresses generation of astrocytes by NSCs derived from the SVZ. A. NSCs derived from the SVZ show a similar profile of differentiation as those derived from spinal cord. β1-integrin Knock-Out (KO) increases astrocytic differentiation (GFAP - red) without altering neuronal (βIIItubulin - green) or oligodendroglial (O4 -red) differentiation. S*cale bar = 20 µm*. B. Western blot analysis shows depletion of β1-integrin protein in the β1KO derived NSCs compared with control. C. Quantification of total cell numbers (DAPI) in the cultures of control and β1-integrin null cells analyzed for lineage commitment (D). There was no difference in overall cell numbers (n = 20, p = 0.91). Values are means ± SEM. D. Quantification of cell numbers of different lineages shows a significant increase (*p≤0.046) in astrocytes (56% increase) in the β1-integrin KO group compared with control at 7DIV without any change in neuronal or oligodendroglial differentiation. E. Quantification of mRNA levels by qPCR after 7DIV reveals a significant increase in GFAP mRNA in the β1-integrin KO group compared with control. βIIItubulin and CNPase mRNAs were unchanged.

To determine whether this regulatory process is unique to the ependymal population of NSCs, we repeated the experiment using SVZ-derived NSCs with strikingly similar results. The total number of cells did not differ between control and β1 integrin null cultures (Figure 2C). Control SVZ-derived NPCs generated 37.3±3.1% astrocytes, 3.4±0.1% neurons, and 3.1±0.5% oligodendrocytes (Figure 2A, 2D). Knockout of β1-integrin (Figure 2B) did not alter neuronal or oligodendroglial lineage differentiation but significantly increased the number of GFAP^+^ astrocytes generated to more than 55% (55.65±7.8%) of the cells (p≤0.046). Knockout of β1-integrin had an even greater effect on levels of GFAP mRNA which increased more than 2-fold compared to control, but levels of βIIItubulin and CNPase mRNAs were unchanged (Figure 2E). Thus the astrocyte-suppressive effects of β1-integrin are comparable in ESCs and in SVZ NSCs (Figure1E, 2D).

### Inhibition of β1 integrin signaling by a dominant negative construct increases astrocytic differentiation

**Figure 3:**
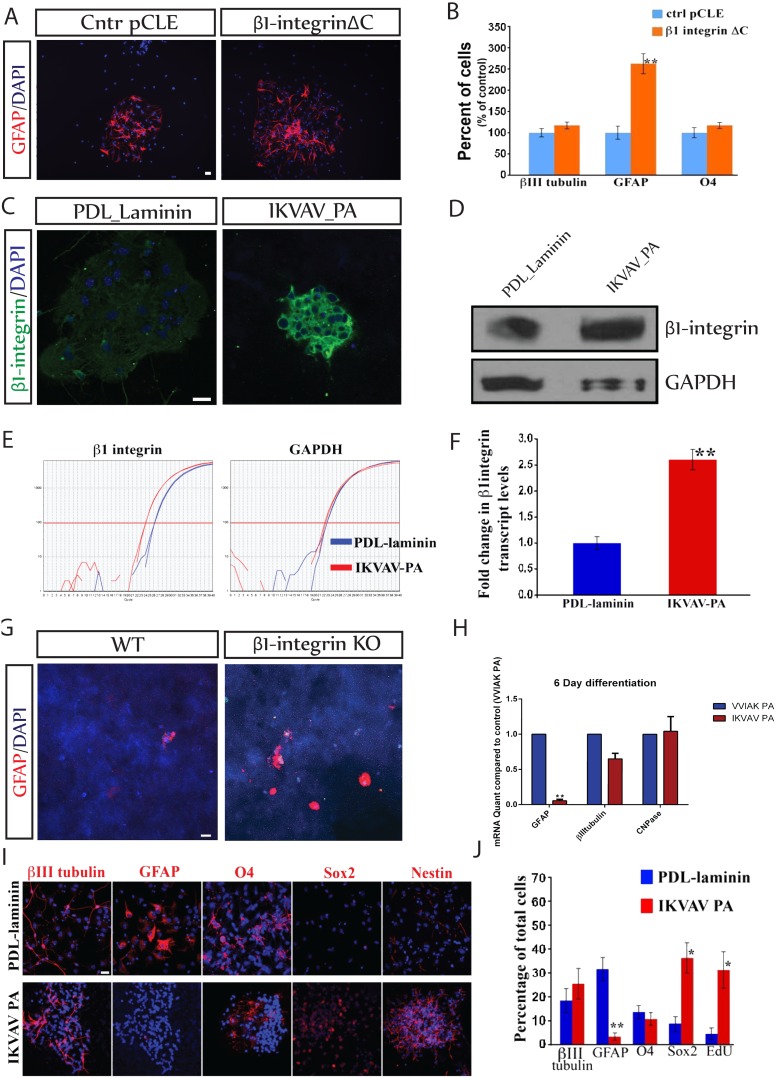
β1-integrin regulates astrocytic differentiation. A. NSCs were infected with retrovirus expressing GFP alone (ctrl pCLE) orβ1-integrinlacking the cytoplasmic domain (β1-integrin ΔC), cultured for 7 days on PDL-laminin and immunostained for GFAP (red) and DAPI (blue). *Scale bar = 20 µm*. B. Astrocytic differentiation (GFAP expression) increased more than 2.5 fold (**p≤0.005) in the β1- integrin ΔC group compared to control while neuronal (βIIItubulin) and oligodendroglial (O4) differentiation were unchanged. C. NSCs were cultured either on PDL-laminin or IKVAV-PA for one day and immunostained with β1- integrin (green) and DAPI (blue). Culture in IKVAV-PA dramatically increased expression of β1- integrin. *Scale bar = 20 µm*. D. Western Blot analysis shows that NSCs express higher levels of β1integrin protein when cultured for one day in IKVAV-PA compared to control (PDL-laminin). E. Real time PCR amplification plots showing that the β1integrin transcripts increase in cells cultured in IKVAV-PA compared to control (PDL-laminin). F. Quantitation of the increase in transcript levels of β1integrin in the IKVAV PA versus PDL-laminin group. (**p≤0.0007). G. KO of β1-integrin increases the number of astrocytes (GFAP – red) generated by NSCs cultured in IKVAV PA for 7 DIV. Blue = DAPI. H. NSCs were cultured in either control PA (VVIAK PA) or IKVAV PA for 7DIV, and RNA was extracted for qPCR quantification. The graph represents each lineage marker mRNA level in IKVAV PA normalized to levels in the control PA. Levels of GFAP mRNA were profoundly decreased (**p≤0.0005 in the IKVAV-PA group) without any significant change in levels of βIIItubulin of CNPase mRNAs. I. NPCs were cultured either on PDL-laminin or in IKVAV PA for 7DIV and immunostained with DAPI (blue) and several lineage markers (red). Note the absence of GFAP staining in the IKVAV PA group and the increase in the progenitor markers Sox2 and nestin. *Scale bar = 20 µm*. J. Quantification of the percentages of cells in the conditions described in (i) (*p≤0.017, **p≤0.005).

After binding to ligands, integrin receptors trigger an intracellular signaling cascade mediated predominantly via a small cytoplasmic domain of the β subunit [12]. This domain can cross talk with multiple intracellular signal transduction systems [28], thereby altering progenitor cell responses to extrinsic factors [13]. Specifically the cytoplasmic domain of β1 integrin mediates the majority of its intracellular signaling [12], and mutant forms of β1 integrin that lack this domain act as a dominant negative form of the receptor [20], [22], [41]. We misexpressed this form of β1 integrin, hereafter referred to as β1ΔC, in NSCs using retroviral mediated gene transfer. Cultured NSCs were transduced with either control pCLE-IRES-GFP or β1ΔC-IRES-GFP retroviruses, FAC-sorted 48 hours later for the positively transfected cells, and plated in differentiating conditions. Seven days after plating, the percentages of neurons (βIII tubulin), astrocytes (GFAP) and oligodendrocytes (O4) were assayed. Expression of β1ΔC increased the number of GFAP^+^ astrocytes more than 2.5-fold (p≤0.005) compared to control pCLE cultures (Figure 3A, 3B), without altering numbers of neurons or oligodendrocytes (Figure 3B). This further supports the conclusion that β1-integrin signaling suppresses astrocytic differentiation.

### IKVAV-PA increases β1-integrin expression in cultured NSCs and suppresses astrocytic differentiation

We next tried to overexpress β1-integrin in cultured NSCs using techniques identical to those used to overexpress β1ΔC. Since the overexpressed cDNA lacked the 3′UTR, we could design PCR primers that detect the endogenous transcript alone as well ones that detect total β1 integrin. At 2 days post infection, there was a significant (∼3 fold) increase in the levels of total β1-integrin. However, even at this time levels of endogenous transcript had already decreased by 60%. At 7 days, we could detect no difference in the levels of β1-integrin between the two groups (not shown). Multiple attempts to overexpress β1-integrin failed, presumably due to a strong feedback loop that maintained β1-integrin expression at a stable level. By contrast, culture of NSCs in the presence of IKVAV-PA markedly increased levels of β1-integrin expression compared to control cells grown in the presence of laminin (Figure 3C, 3D, 3E, 3F). There were significant increases in expression of both β1-integrin protein (Figure 3C, 3D) and mRNA (Figure 3E, 3F).

We also evaluated the expression of different integrin subunits in cultured NSCs and found significant levels of α5, α6, and αV but no significant levels of β3 or β4 integrins. We then examined the levels of these integrin transcripts in NSCs cultured in IKVAV PA for 7 days using real time RT-PCR. There was a significant (∼3 fold) increase in the β1 integrin transcript levels in cells cultured in IKVAV PA as compared to the control (Figure 3E, 3F). However, there were no changes in levels of the α5, α6 or αV subunits and no detectable levels of the β3 or β4 subunits. This suggests that IKVAV-PA is a uniquely effective tool for increasing β1-integrin expression by NSCs.

Culture of NSCs in the presence of IKVAV-PA increased levels of β1-integrin expression and suppressed astrocytic differentiation suggesting a causal relationship [47]. However, to directly test whether the suppressive effects on astrocyte development were due to the expression of β1-integrin, we examined the effects of IKVAV-PA on NSCs in which β1-integrin was knocked out using Adeno-Cre retrovirus as described above (Figure 3G). Culture of control NSCs in IKVAV-PA markedly suppressed astrocytic differentiation (12.5±1.4% of cells) as previously described [48]. However, more than 37% of cells (37.3±5.1%) in which β1-integrin was ablated differentiated into GFAP^+^ astrocytes even in the presence of IKVAV-PA. Thus the suppressive effects of IKVAV-PA on astrogliosis depended upon the presence of β1-integrin in the cells. We next wanted to ascertain that the effects of IKVAV-PA depended upon the integrity of the IKVAV epitope and were not related to other physicochemical properties of the PA. We therefore compared the effects of IKVAV-PA on mRNA expression with those of a PA in which the amino acid sequence of the epitope was scrambled (VVIAK-PA) (Figure 3H). The profound inhibitory effects of IKVAV-PA on expression of GFAP mRNA were lost when the IKVAV epitope was scrambled. However, there were no significant differences in levels of either βIIItubulin or CNPase mRNAs in IKVAV-PA and VVIAK-PA treated cells, indicating the specificity of the effects of the IKVAV epitope on expression of GFAP mRNA.

IKVAV-PA consistently suppressed astrocyte differentiation by cultured NSCs without changing either neuronal or oligodendroglial differentiation. This suggested that the PA maintained cells in an undifferentiated stem/progenitor cell state. To directly test this hypothesis we examined the effects of the PA on expression of the NSC marker, SOX2, as well as incorporation of the thymidine analog EdU. We used the most optimal substratum for control cells, PDL-laminin, for comparison even though it presented to cells a small amount of the IKVAV epitope (Figure 3I, 3J). Again the IKVAV-PA almost completely suppressed astrocytic differentiation (3.2±0.8% of the cells expressed GFAP versus 32.4±4.7% in controls; p≤0.005) without altering neuronal (βIIItubulin) or oligodendroglial (O4) lineage differentiation. However, the decrease in the percentage of GFAP-expressing cells was almost exactly matched by an increase in the percentage of cells that expressed Sox2 and that were proliferative as measured by EdU incorporation. These observations indicate that IKVAV suppressed astrocytic differentiation by maintaining cells in the NSC state.

### The ILK pathway mediates suppressive effects of β1-integrin on astrocytic differentiation

**Figure 4:**
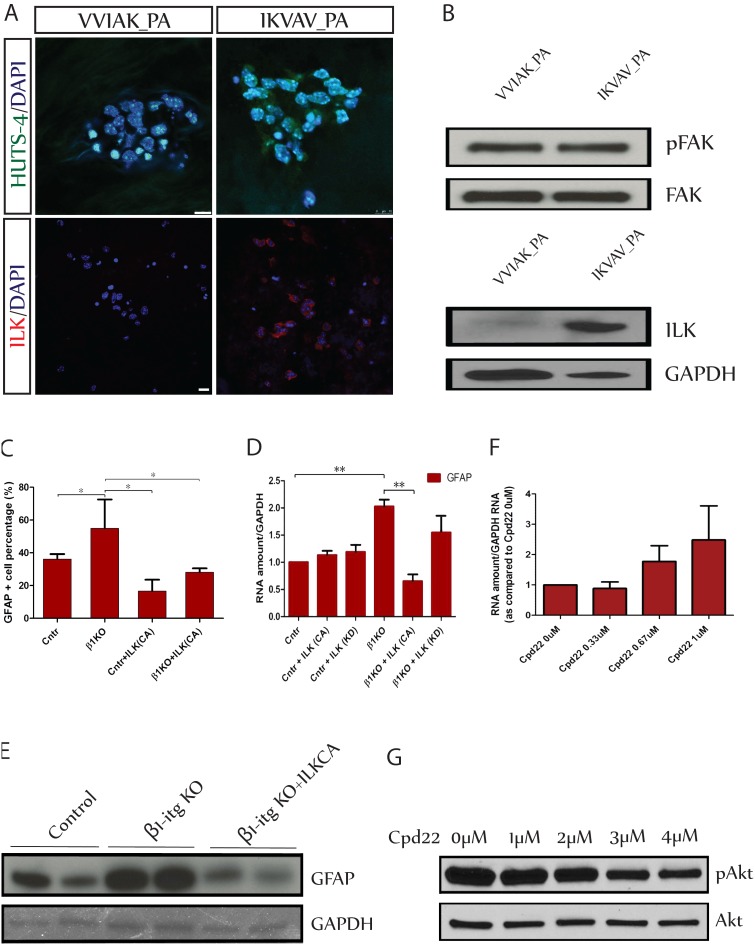
Integrin Linked Kinase (ILK) regulates astrocytic differentiation by NSCs. A. NSCs cultured in either control (scrambled) PA (VVIAK PA) or IKVAV PA for one day, and immunostained with activated β1integrin antibody HUTS-4 (green) or ILK (red). *Scale bar in HUTS-4 staining image = 10 µm, and in the ILK staining image = 20 µm.* B. Western blot analysis of NSCs cultured in either control PA (VVIAK PA) or IKVAV PA for one day. Note that levels of ILK are markedly elevated in the IKVAV-PA group without any change in FAK. C. β1-integrin KO and control NSCs were transfected with either a control construct or a construct expressing a constitutively active form of ILK (ILK-CA). At 7DIV the cells were immunostained for GFAP and numbers of GFAP^+^ cells were counted. (*p≤0.05) D. β1-integrin KO and control NSCs were transfected with a control construct, a construct expressing constitutively active ILK (ILK-CA), or a construct expressing kinase-dead ILK (ILK-KD) and cultured for 7DIV. RNA was extracted for qPCR measurement of GFAP mRNA graphed as a ratio compared to the control (**p≤0.004). Note that β1-integrin KO increased levels of GFAP mRNA, but this increase was blocked by expression of ILK-CA. E. Western blot analysis of GFAP expression in control NSCs and in β1-integrin KO NSCs transfected with either with a control construct or ILK-CA. Note that transfection with ILK-CA prevented the increase in GFAP expression in β1-integrin KO NSCs. F. NSCs were treated with different doses of the ILK inhibitor, Cpd22. After 6DIV RNA was extracted for qPCR quantification of GFAP. Note that GFAP expression increased in a dose dependent manner after treatment with Cpd22. H. NSCs were treated with different doses of the ILK inhibitor, Cpd22 for 3h, and protein was extracted for western blot analysis. Note that treatment with the drug reduced levels of phospho-Akt without any change in levels of AKT.

We next sought to determine whether IKVAV-PA actually activates β1-integrin and to define the signaling pathways mediating the effects of β1-integrin on astrocytic development by NSCs. Integrins are heterodimeric receptors, and the relationship between the α and β subunits changes after ligand binding resulting in an “activated” protein conformation [29]. This conformational change initiates intracellular signaling and also increases the affinity of ligand binding [9], [52]. We used a conformation sensitive β1-integrin antibody, HUTS-4 [29], to detect the activated form of β1-integrin in NSCs one day after plating in either IKVAV-PA or VVIAK-PA (Figure 4A). Almost all cells cultured in IKVAV-PA immunostained for the activated conformation of β1-integrin whereas little immunostaining was detectable in cells culture in VVIAK-PA. This provides evidence that IKVAV-PA rapidly activated β1-integrin.

β1-integrin signals through a variety of different mechanisms including but not limited to associating with other membrane proteins, serving as a binding dock for intracellular signaling molecules to bind and aggregate, and activating a specific set of intracellular domain bound kinases such as focal adhesion kinase (FAK) and integrin-linked kinase (ILK) [1], [5]. Integrin-linked kinase (ILK) is a 59kDa serine/threonine protein kinase that associates with the cytoplasmic domain of β-integrins and transduces signaling after activation of the integrin [17]. Western blot analysis showed no difference in levels of FAK in NSCs grown in IKVAV PA compared to cells grown in VVIAK PA (Figure 4B). By contrast, culture of the cells in IKVAV PA resulted in a very large increase in levels of ILK (Figure 4B). Similarly, immunostaining for ILK indicated that most NSCs cultured in IKVAV PA expressed ILK whereas little staining was apparent in cells cultured in VVIAK PA (Figure 4A).

This suggested that ILK might mediate suppressive effects of β1-integrin on astrocytic development. To explore this hypothesis, we designed a rescue experiment to determine whether expression of a constitutively active form of ILK was sufficient to block the effects of knockout of β1-integrin (Figure 4C). We created two functionally mutated ILK expression constructs based on the findings of Hannigan, Troussard et al [16]. We substituted serine 343 of the potential autophosphorylation site with aspartate (S343D), to get a constitutive active form of ILK (ILK_CA), and we substituted serine 343 with Alanine (S343A) to make a kinase-dead ILK (ILK_KD). NSCs were isolated from β1-integrin^flx/flx^ mice and β1-integrin was knocked out as described above by infecting cells with Adeno-Cre or control virus. The cells were electroporated with either the ILK_CA or the ILK_KD construct, FAC- sorted two days later, and plated on PDL-Laminin for 7 days in differentiating conditions. On day 7 cells were examined immunocytochemically (Figure 4C) and also were examined for levels of mRNAs for βIIItubulin, CNPase, and GFAP (Figure 4D). There were no detectable differences in the number of either βIIItubulin or CNPase immunoreactive cells across the different conditions (not shown). 37.3±3.1% of control cells expressed GFAP, but knockout of β1-integrin increased the percentage of GFAP-immunoreactive cells to 55.65±7.8% (p≤0.05) (Figure 4C). Overexpression of ILK-CA reduced the percentage of GFAP^+^ cells to 16.7±4.0%, and overexpression of ILK-CA in β1-integrin knockout cells prevented the increase associated with knockout of β1-integrin (GFAP^+^ cells number dropped to 28.1±1.4%). There were no detectable differences in levels of mRNA encoding either βIIItubulin or CNPase across the different conditions (not shown). Moreover neither construct exerted any effect on GFAP mRNA in control cells. However, knockout of β1-integrin increased levels of GFAP mRNA more than twofold, but expression of the ILK-CA construct prevented this increase and levels of mRNA in these cells actually trended lower than in control cells (Figure 4D). We then examined levels of GFAP protein in these cells by western blot analysis. Knockout of β1-integrin resulted in a very large increase in levels of GFAP protein (Figure 4E). Expression of the ILK-CA construct in β1-integrin knockout cells not only prevented the increase in GFAP but actually reduced it below control levels. There were no differences in proliferation (EdU^+^ incorporation) among the groups. These observations indicate that ILK signaling suppresses astrogliogenesis, analogous to the effects of β1-integrin, and suggest that ILK mediates at least some of the effects of β1-integrin on astrocytic differentiation. To further test this hypothesis, we examined the effects of a chemical inhibitor of ILK, Cpd22 [23], on differentiation of NSCs. ILK has been shown act directly on Ser473 kinase in Akt [19]. To first verify that the inhibitor actually acted as expected in NSCs, the cells were treated with different concentrations of Cpd22 one day after plating (Figure 4G). 3 hours after drug application, we harvested protein for western blot analysis and found a dose-dependent inhibition of levels of phospho-Akt without any change in the overall levels of Akt. This suggested that the drug was able to penetrate NSCs and exert effects on a known ILK target. We therefore next assessed the effects of Cpd22 treatment on astrocytic differentiation. We chose a dose range that was tolerated well by the cells for a 6 day period of treatment without any evidence of cell death and examined the cells for levels of GFAP mRNA. Treatment of NSCs with the inhibitor resulted in a dose-dependent increase in levels of GFAP mRNA (Figure. 4F). These data are supportive of the hypothesis that ILK signaling inhibits astrocytic differentiation.

### IKVAV PA activates β1-integrin and suppresses glial scar formation after SCI

**Figure 5:**
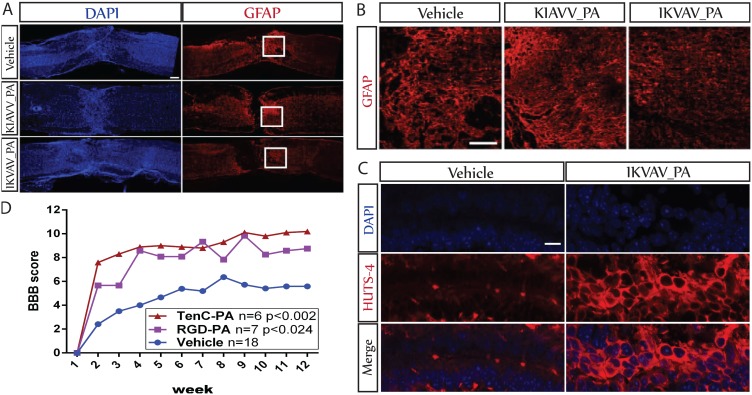
β1-integrin signaling in vivo regulates astrogliogenesis after spinal cord injury (SCI). A. Low magnification (10X) images of representative longitudinal sections of injured mouse spinal cords at 3 weeks post SCI, that were injected with either the IKVAV PA, scrambled KIAVV PA or vehicle injected at 24 hours post injury. Sections are stained with DAPI (blue) and GFAP (red). *Scale bar = 200 µm.* B. Representative confocal Z-stacks taken at higher magnification (20X) of the areas boxed in (a) showing reduced gliosis in the IKVAV PA injected animals at 3 weeks post SCI. *Scale bar = 100 µm.* C. High magnification (63x) confocal images of the ependymal regions stained with activated β1integrin antibody HUTS-4 (red) and DAPI (blue). Injured mouse spinal cords were injected either with IKVAV PA or vehicle 2 days post injury. *Scale bar = 20 µm.* D. Spinal cord injured rats were injected with either Tenascin-C epitope presenting PA, RGD epitope presenting PA or vehicle 2 days post injury, and monitored with BBB scoring for 12 weeks. Both PA groups showed significant improvement compared to the vehicle group.

Previously we reported that injection of IKVAV-PA into an injured spinal cord suppresses glial scar formation and enhances behavioral outcome [54]. Since our findings *in vitro* suggested that IKVAV-PA suppresses astrocytic development by activating β1-integrin, we sought to determine whether the PA actually activates β1-integrin signaling in the injured spinal cord. IKVAV-PA and a PA with a bioinert epitope (KKIAV-PA) were injected into the damaged spinal cord 24 hours after a contusion injury. The spinal cords were removed 3 weeks later for immunocytochemical analyses. As previously reported, injection of IKVAV-PA significantly reduced glial scarring as indicated by GFAP immunocytochemistry (Figure 5A, 5B). By contrast, the PA with an inert epitope had no demonstrable effect.

We then examined the spinal cords immunocytochemically using the HUTS-4 antibody to probe for the activated conformation of β1-integrin. IKVAV-PA and control vehicle were injected into the damaged spinal cord 48 hours after a contusion injury. The spinal cords were removed 1 week later for immunocytochemical analyses. Very little HUTS-4 staining was detectable in a vehicle-injected spinal cord (Figure 5C). By contrast, there was abundant HUTS-4 staining in the spinal cord injected with IKVAV-PA, demonstrating that the PA activated β1-integrin signaling *in vivo*.

Injection of IKVAV-PA into the injured spinal cord also consistently improved behavioral outcome [54] as measured by open field testing [3], [21], but it was unclear whether the behavioral enhancement was also due to β1-integrin signaling. To help determine this we examined the effects of PAs displaying two different epitopes that are known to interact with β1-integrin, a Tenascin C epitope and the fibronectin epitope RGD [39], [55]. The Tenascin C epitope, ADEGVFDNFVLK, is just a small portion of Tenascin C that interacts specifically with β1-integrin [31], [33]. We injected these PAs into injured spinal cords of 8 weeks old adult rats two days after a standard impaction injury and followed behavioral outcome for 12 weeks. Similar to prior findings with IKVAV-PA, we found improvements with both Tenascin C-PA and RGD-PA (Figure 5D). Vehicle-injected animals reached an average score of 5.58±0.59 by 12 weeks after the injury. The Tenascin C-PA group reached an average score of 10.20±0.84 (p≤0.002) and the RGD-PA group achieved an average score of 8.5±2.1 (p≤0.024).

## Discussion

β1-integrin is a transmembrane receptor protein expressed by stem cells in many organ systems [15], [25]. β1-integrin signaling mediates a variety of stem cell functions including pluripotency maintenance, self-renewal, proliferation and regulation of migration [25]. In the present study, we report that β1-integrin signaling inhibits astrocytic differentiation by both spinal cord ependymal stem cells and subventricular zone stem cells and helps to maintain stemness. We further found that these effects are mediated, at least in part, by ILK. In previous studies we found that injection of IKVAV-PA into an injured spinal cord limited glial scar formation and enhanced behavioral outcome. In this present study, we report an underlying mechanism to be a ligand (IKVAV) mediated increase of β1 integrin signaling in ependymal cells which suppresses astrogliosis.

Astrogliosis after SCI has both beneficial and detrimental effects on recovery. Astrocytic hypertrophy is necessary for repairing the damaged blood-brain barrier, and it is a beneficial process that limits inflammatory damage and restores homeostasis [10], [27], [38]. However, astrocytic hyperplasia leads to formation of a dense glial scar that inhibits axonal regeneration [48]. Therapeutic approaches to limiting glial scar formation after SCI must therefore seek to limit the detrimental effects of astrocytic hyperplasia while maintaining the beneficial effects of the initial reactive astrocytes. In previous studies we found that injection into an injured spinal cord of IKVAV-PA limited astrocytic hyperplasia without altering the early hypertrophic response to the injury [54]. However, the reason for these divergent effects was unclear. In the present study we found that ESCs expressed high levels of β1-integrin after SCI whereas levels of the protein were not demonstrably increased in resident astrocytes after the injury. Further, our findings suggested that the effects of the PA reflected limitation of ESC differentiation into astrocytes. By contrast, β1-integrin signaling does not alter astrocyte proliferation although it exerts effects on reactive gliosis [43]. Thus injection of IKVAV-PA into a damaged spinal cord was able to limit astrogliosis by limiting ESC differentiation into astrocytes without altering the beneficial responses of resident astrocytes to the injury. Our findings indicate a causal relationship between IKVAV PA application and β1-intregrin signaling activation in vivo. This conclusion is supported by our observation that ablation of β1-intregrin in ESCs significantly worsens behavioral outcome after SCI (unpublished observation).

Identification of the molecular mechanisms underlying the beneficial effects of the PA should enable design of new targeted molecules that are even more effective in promoting recovery after SCI. For example, we found that PAs displaying two other epitopes known to interact with β1-integrin, a Tenascin C epitope and the RGD epitope of fibronectin, facilitated behavioral recovery after SCI. Ultimately it should be possible to design molecules that are even more effective therapeutically. For example, PAs can be created that incorporate more than one β1-integrin-interacting epitope and that also include epitopes targeting other signaling molecules such BMP receptors [44] or cytokines that activate JAK-STAT signaling [37] that have been implicated in astrocytic responses after SCI.

The increase in GFAP expression after ablation of β1-integrin in NSCs was accompanied by an increase in the number of GFAP^+^ astrocytes detected by immunocytochemistry suggesting an increase in astrocyte lineage commitment. Because this comparison was done under differentiation conditions with very low levels of EGF, proliferation was limited. However it is possible that the increase in GFAP expression simply allowed detection of preexisting astrocytes with previously undetectable levels of GFAP. In either case, our findings support our hypothesis that β1-integrin signaling suppresses astrocytic differentiation, and is consistent with our finding *in vivo* that increased β1-integrin signaling leads to reduced glial scar formation. It should also be noted that the increase in GFAP^+^ cells *in vitro* in the presence of IKVAV-PA was matched by a decrease in the number of Sox2^+^ stem cells suggesting an effect on lineage commitment.

Our observations indicate that at least some of the effects of β1-integrin signaling on astrocytic differentiation are mediated by ILK, but the precise mechanisms mediating the effects of ILK remain unclear. ILK is a serine/threonine protein kinase that directly affects numerous other signaling pathways [17], [24]. For example, in dendritic formation ILK directly phosphorylates GSK-3beta thus inhibiting its signaling [35]. However, in addition to its kinase activity, ILK can initiate signal transduction through its function as a scaffolding protein. For example, in mouse kidney development, the auto-phosphorylation site of ILK is dispensable, whereas the alpha-parvin binding ability of ILK is critical for normal development [49]. In both cases the effects of ILK reflect modulation of other important signaling pathways in the cell. Possible candidates include the PI3K/Akt and PI3K/RhoA pathways, that are known downstream targets of ILK and that have been implicated in reducing the potential for astrocytic differentiation [6]. However in other systems ILK signaling has also been linked to BMP and JAK-STAT signaling which are the major pathways involved in astrocytic differentiation [26], [51]. Delineation of the set of pathways that mediate the effects of ILK on astrogliosis will ultimately be required to design effective interventions for limiting gliosis after injury to the nervous system.

It is noteworthy that the effects of β1-integrin signaling on astrocytic development were almost identical in ESCs isolated from the adult spinal cord and NSCs isolated from the adult SVZ. In turn this suggests that this signaling system may be an important target for regulating astrogliosis after brain injury as well as after SCI. Although this study did not examine the responses of subgranular zone stem cells to β1-integrin signaling, we find that the molecule is expressed abundantly in the SGZ. Thus it is likely that β1-integrin signaling an important regulator of stem cell proliferation and differentiation throughout the adult nervous system.
